# An Effective and Feasible Method, “Hammering Technique,” for Percutaneous Fixation of Anterior Column Acetabular Fracture

**DOI:** 10.1155/2016/7151950

**Published:** 2016-07-14

**Authors:** Lihai Zhang, Peng Yin, Wei Zhang, Tongtong Li, Jiantao Li, Hua Chen, Qi Yao, Peifu Tang

**Affiliations:** ^1^Department of Orthopaedics, Chinese PLA General Hospital, No. 28 Fuxin Road, Beijing 100853, China; ^2^Medical College, Nankai University, No. 94 Weijin Road, Tianjin 300071, China; ^3^Department of Orthopaedics, Beijing Shijitan Hospital, Beijing 10038, China

## Abstract

*Objective*. The objective of this study was to evaluate the effectiveness and advantages of percutaneous fixation of anterior column acetabular fracture with “hammering technique.”* Materials and Methods*. We retrospectively reviewed 16 patients with percutaneous fixation of anterior column acetabular fracture with “hammering technique.” There were 11 males and 5 females with an average age of 38.88 years (range: 24–54 years) in our study. Our study included 7 nondisplaced fractures, 6 mild displaced fractures (<2 mm), and 5 displaced fractures (>2 mm). The mean time from injury to surgery was 4.5 days (range: 2–7 days).* Results*. The average of operation time was 27.56 minutes (range: 15–45 minutes), and the mean blood loss was 55.28 mL (range: 15–100 mL). The mean fluoroscopic time was 54.78 seconds (range: 40–77 seconds). The first pass of the guide wire was acceptable without cortical perforation or intra-articular perforation in 88.89% (16/18) of the procedures, and the second attempt was in 11.11% (2/18).* Conclusion*. Our study suggested that percutaneous fixation of anterior column acetabular fracture with “hammering technique” acquired satisfying surgical and clinical outcomes. It may be an alternative satisfying treatment for percutaneous fixation of anterior column acetabular fracture by 2D fluoroscopy using a C-arm with less fluoroscopic time.

## 1. Introduction

The incidence of acetabular fractures is 3 per 100,000 inhabitants per year [1]. Anterior column fractures account at least for 12.3% of all acetabular fractures [2]. The treatment of displaced acetabular fractures with open reduction and internal fixation has become the standard method [3–7]; however, extensive exposure may be complicated by blood loss, infection, neural or vascular injury, heterotopic ossification, and wound healing problems [8–10]. Recently, percutaneous fixation with different techniques of visualization has successfully been used for the management of displaced or nondisplaced acetabular fractures [3, 11–13]. Percutaneous fixation of acetabular fractures is associated with fewer complications compared with open procedures, especially in patients with multiple medical problems [11, 13]. Therefore, percutaneous fixation of acetabular fractures as a minimally invasive procedure has gradually gained general acceptance [3, 11].

However, percutaneous periacetabular screw placement is a demanding procedure due to the narrow osseous corridors and complex acetabular anatomy [6, 11]. The anterior column screw as a type of periacetabular screws could be placed in either antegrade or retrograde direction, but either of which is a demanding technique, especially in some female patients [6, 7, 14, 15]. Chen et al. reported that the anterior column of acetabulum could accommodate 6.5 mm lag screw very well in all the males, but the intraosseous space of anterior column in 22.62% females was smaller than 6.5 mm [14]; therefore, the osseous corridor of anterior column is narrower in some females.

As we know, the anterior column of acetabular is composed of cortical and cancellous bone, and the cancellous bone is situated in the middle of the anterior column. Therefore, the entering process of the guide wire in the anterior column could be judged by the “hands' feeling,” and we performed a technique of knocking the guide wire lightly by a hammer through the narrow osseous corridor in order to decrease the rates of screw perforation. In the following report, we described our successful experience about the technique.

## 2. Patients and Methods

From January 2010 to January 2011, 26 patients with anterior column acetabular fractures were treated in our institution. Our inclusion criteria were (1) patients with a nondisplaced or mild displaced fracture (2 mm or less) or displaced fracture that could be reduced with a closed or limited open approach; (2) patients of age of 18 years or more; (3) the injured acetabulum being normal before injury. The exclusion criteria were (1) patients with an open or pathological fracture; (2) patient being complicated by serious nerve or vascular injury. According to the inclusion and exclusion criteria, 16 patients with 18 anterior column acetabular fractures were included in our study. Our study was approved by the Chinese PLA General Hospital committee for clinical research and informed consent was obtained from the 16 patients.

There were 11 males and 5 females with an average age of 38.88 years (range: 24–54 years) in our study. The mechanism of injury included traffic accident in 12 patients and falling in 4 patients. The right acetabular fractures were found in 7 patients, and the left acetabular fractures were found in 7 patients and the rest were 2 bilateral acetabular fractures. Our study included 7 nondisplaced fractures, 6 mild displaced fractures (<2 mm), and 5 displaced fractures (>2 mm). The mean time form injury to surgery was 4.5 days (range: 2–7 days). More details were listed in Table 1.

## 3. Surgical Technique

**Figure 1:**
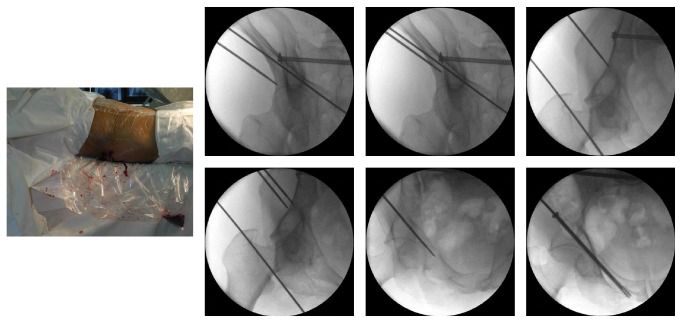
The anterior column screw was placed in an antegrade direction.

**Figure 2:**
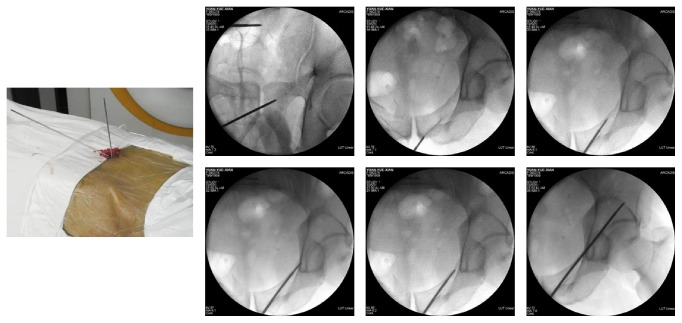
The anterior column screw was placed in a retrograde direction.

**Figure 3:**
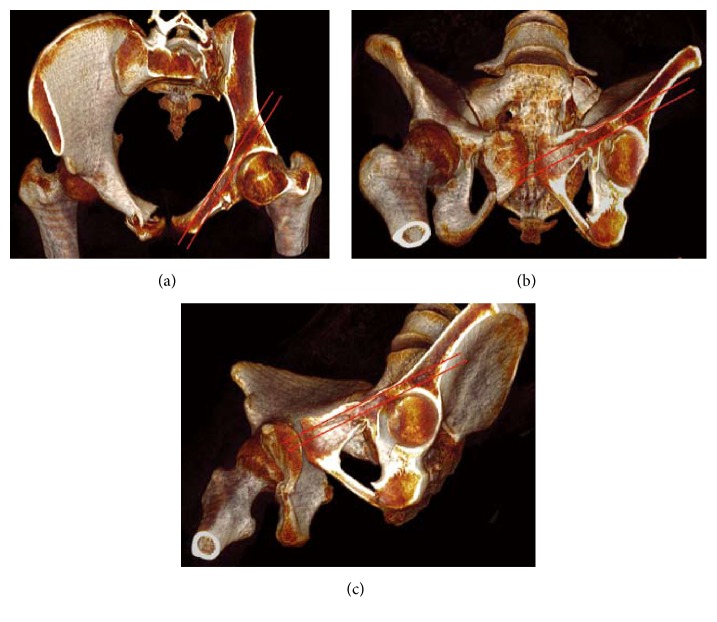
The schematic diagram of the corridor of anterior column acetabular screw. (a) Pelvic inlet; (b) pelvic outlet; (c) obturator outlet.

The patient was positioned supine on a radiolucent table under general anesthesia. 2D fluoroscopy using a C-arm was employed to confirm satisfactory reduction and monitor the safety and accuracy of passage of an initial guide wire. For displaced fractures, reduction was performed first via a closed approach; if the reduction was not acceptable, the fractures were reduced through a limited approach with the aid of a reduction clamp [16]. There were two ways for screw placement in our study. For antegrade placement, the direction was from the eminence of gluteus medius above the acetabulum toward ipsilateral pubic tubercle (Figure 1). For retrograde placement, the direction was opposite to the antegrade placement (Figure 2). After obtaining anatomical reduction, the optimal entry point was chosen by fluoroscopic images, including pelvic inlet, outlet, Judet, and anterior-posterior views. One cm skin incision was performed in order to expose the site of entry point, and then 2.8 mm hard elastic guide wire was knocked into the bone lightly by a hammer from the entry point. The guide wire should not be vertical to the surface of bone in the entry point in order to avoid penetrating the osseous corridor. The entering process of the guide wire was usually judged by the “hands' feeling,” but if surgeon was not sure whether the guide wire penetrates the osseous corridor or not, 2D fluoroscopy using a C-arm would be employed to confirm the result. Therefore, the surgeons should be familiar with the corridor of anterior column of anterior column acetabular screw, which was shown in Figure 3, and they also should have some clinical experience of the “feeling” that the guide wire is placed in the intraosseous space. When the guide wire reached the site of exit point, fluoroscopic image was performed to confirm the safety of osseous corridor. If the confirmatory image was satisfying, a proper cannulated screw would be passed over the guide wire. Finally, the incision was closed.

## 4. Postoperative Care

The continuous passive motion was initiated on the first postoperative day. Assisted active range of motion and isometric exercises were started at the second day after the operation. Progressive weight bearing was started at 10–12 weeks after the operation.

## 5. Evaluation of Outcome

Operation time, blood loss, fluoroscopic time, and the numbers of attempts of guide wire were recorded. The final clinical outcomes of patients were evaluated by Harris Hip Score [17, 18]. The outcomes were categorized as excellent (90–100 points), good (80–89 points), fair (70–79 points), or poor (<70 points).

## 6. Results

The average of operation time was 27.56 minutes (range: 15–45 minutes), and the mean blood loss was 55.28 mL (range: 15–100 mL). The mean fluoroscopic time was 54.78 seconds (range: 40–77 seconds). The first pass of the guide wire was acceptable without cortical perforation or intra-articular perforation in 88.89% (16/18) of the procedures, and the second attempt was in 11.11% (2/18). All the patients were followed up, and the average time of follow-up was 29.06 months (range: 18–40 months). The mean points of Harris Hip Score were 88.83 (range: 79–95 points). According to the criteria of Harris Hip Score, there were 10 clinical outcomes rated as excellent, 7 outcomes rated as good, and 1 outcome rated as fair. More details were listed in Table 2. There were no serious complications in our study.

## 7. Discussion

The present study showed that the management of anterior column acetabular fractures of displaced or nondisplaced by percutaneous fixation acquired satisfying clinical outcomes. The first pass of the guide wire was acceptable in 88.89% (16/18) of the procedures, and the excellent and good rate of clinical outcomes were 94.44% (17/18). There were no serious complications in our study.

The main treatment goal of acetabular fractures is to restore the normal shape of the acetabulum to prevent postoperative traumatic osteoarthritis and allow early weight bearing to rehabilitate function [3, 7]. Various treatments have been applied to the management of acetabular fractures successfully, including conservative treatment, open reduction and internal fixation, and percutaneous fixation. Conservative treatment has been used for the management of nondisplaced or mild fractures successfully, but long term immobilization and inadequate fixation can cause various complications, such as joint stiffness, pulmonary and urinary infections, and unstable fracture redisplacement [7, 19, 20]. Open reduction and internal fixation require extensive exposure, which may be complicated by blood loss, infection, neural or vascular injury, heterotopic ossification, and wound healing problems [8–10]. Percutaneous fixation is a minimally invasive technique, and it can provide satisfying clinical outcomes with less related complications [3]. However, the osseous corridor for screw placement usually is very narrow, and screw perforation sometimes happens. Therefore, we performed a technique of knocking the guide wire lightly by a hammer through the narrow osseous corridor in order to decrease the rates of screw perforation.

In our study, the first pass of the guide wire was acceptable in 88.89% (16/18) of the procedures, which is better than the 83.3% reported by Crowl and Kahler [3]. The excellent and good rate of clinical outcomes were equal to the 94% reported by Crowl and Kahler [3]. We performed the procedures by 2D fluoroscopy using a C-arm, and we believe that it is good for applying in these hospitals without equipping CT navigation. The mean fluoroscopic time was 54.78 s in our study, which is lower than the 62 s reported by Kaempffe et al. [7] and the 73 s reported by Vioreanu and Mulhall [21]. The average of operation time was 27.56 minutes in our report, which is less than the 30 minutes by Mouhsine et al. [7] and 75 minutes reported by Mouhsine et al. [15]. These results revealed that our technique was easier to perform as long as you had enough clinical experience of the “feeling” that the guide wire “walks” in the passage made up of cancellous bone. The mean blood loss was 55.28 mL in our study, which is lower than the 99 mL reported by 99 mL Magu et al. [11] and a little higher than the 50 mL reported by Mouhsine et al. [15]. Meanwhile, there were no serious complications in our study.

In our experience, some important aspects should be paid attention to: (1) the guide wire should be knocked lightly by a hammer and the guide wire should not be vertical to the surface of bone in entry point; (2) high anterior column acetabular fractures are more easily managed in an antegrade fashion, and low anterior column fractures are more suitable in a retrograde fashion; (3) preoperative Judet films and CT scans are necessary to understand the nature of anterior column acetabular fracture; (4) a wire can be firstly placed in the body surface as a reference to the guide wire to confirm the place of acetabular anterior column, and then the guide wire is knocked into the anterior column in a way that is parallel to the wire (Figure 1).

We described a successful alternative technique for anterior column acetabular fractures through percutaneous fixation. A number of data on the characteristics of patients, clinical data, and experience were reported in our study. However, our study is retrospective in nature and the number of patients is relatively small, and all operations were performed by two senior orthopaedic surgeons who perhaps have a preference in the way of screw placement. There is no control group to compare our results with. More prospective randomized controlled trials are needed to overcome the limitations of our study.

In conclusion, our study suggested that percutaneous fixation of anterior column acetabular fracture by “hammering technique” acquired satisfying clinical outcomes. It may be an alternative satisfying treatment for percutaneous fixation of anterior column acetabular fracture by 2D fluoroscopy using a C-arm with less fluoroscopic time.

## Figures and Tables

**Table 1:** Demographic characteristics of 16 patients with anterior column acetabular fracture.

Case number	Sex	Age (years)	Mechanism of injury	Injured acetabulum	Type of fractures	The time from injury to surgery (days)
1	Male	30	TA	Left	NF	5
2	Female	36	TA	Right	MF	4
3	Male	24	TA	Left	NF	3
4	Male	43	TA	Right	DF	4
5	Female	48	F	Right	NF	7
6	Male	54	TA	Bilateral	NF, MF	2
7	Male	50	F	Left	MF	3
8	Female	37	TA	Right	DF	5
9	Male	28	TA	Left	DF	6
10	Male	37	TA	Right	NF	4
11	Male	28	TA	Left	MF	4
12	Female	45	F	Right	MF	7
13	Male	34	TA	Left	NF	5
14	Male	54	F	Right	MF	4
15	Female	41	TA	Bilateral	NF, DF	3
16	Male	33	TA	Left	DF	6

TA: traffic accident; F: falling; NF: nondisplaced fracture; MF: mild displaced fractures; DF: displaced fracture.

**Table 2:** Clinical data of 16 patients with anterior column acetabular fracture.

Case number	Operation time (minutes)	Blood loss (mL)	Fluoroscopic time (seconds)	The number of attempts of guide wire	Follow-up (months)	Harris Hip Score	Outcomes
1	15	15	40	1	18	95	Excellent
2	22	35	48	1	24	93	Excellent
3	28	55	55	1	22	91	Excellent
4	32	70	62	1	26	88	Good
5	24	40	52	1	30	90	Excellent
6	24, 30^*∗*^	45, 60^*∗*^	51, 58^*∗*^	1, 1^*∗*^	36	83, 86^*∗*^	Good, good^*∗*^
7	24	45	51	1	28	90	Excellent
8	35	80	70	1	30	85	Good
9	32	70	65	2	26	88	Good
10	15	20	42	1	30	94	Excellent
11	30	65	53	1	32	85	Good
12	25	45	48	1	34	90	Excellent
13	26	45	55	1	32	93	Excellent
14	23	50	46	1	28	91	Excellent
15	30, 45^*∗*^	65, 100^*∗*^	55, 77^*∗*^	1, 2^*∗*^	40	90, 88^*∗*^	Excellent, good^*∗*^
16	36	90	58	1	29	79	Fair

^*∗*^This data represent left and right acetabular, respectively.
